# A cecal lesion hiding a secret: underwater endoscopic mucosal resection revealed an occult appendiceal neuroendocrine tumor

**DOI:** 10.1055/a-2830-5028

**Published:** 2026-06-02

**Authors:** Rossella Maresca, Tommaso Schepis, Giulia Piccirilli, Tommaso Rozera, Silvia Pecere, Cristiano Spada, Federico Barbaro

**Affiliations:** 1 Digestive Endoscopy Unit 18654 Fondazione Policlinico Universitario Agostino Gemelli IRCCSRomeItaly; 2 Center for Endoscopic Research Therapeutics and Training (CERTT)Università Cattolica del Sacro CuoreRomaItaly

Endoscopic resection of periappendiceal lesions is technically challenging due to anatomical constraints, limited scope maneuverability, and procedural risks 1 .

We report a case of a 49-year-old woman referred to our unit for the management of a 35-mm non-granular laterally spreading tumor (elevated type) of the cecum. Previous biopsies showed a tubular adenoma with low-grade dysplasia.

**Fig. 1:**
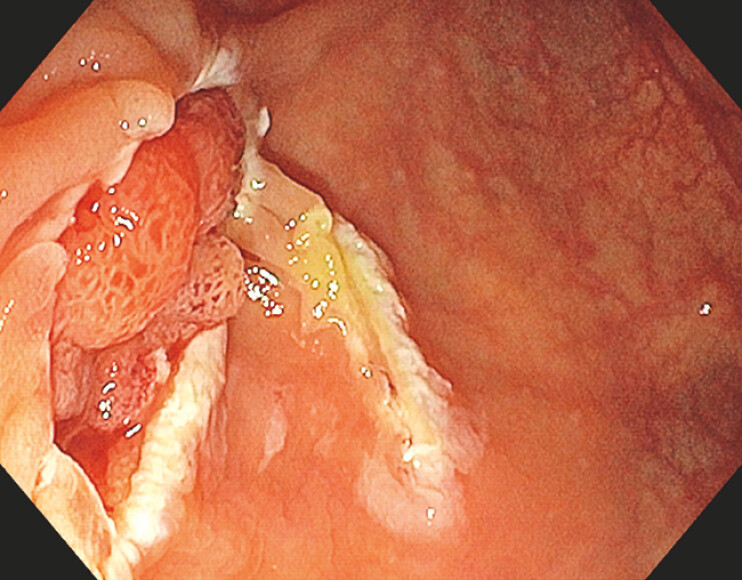
Residual tissue within the appendiceal stump.

**Fig. 2:**
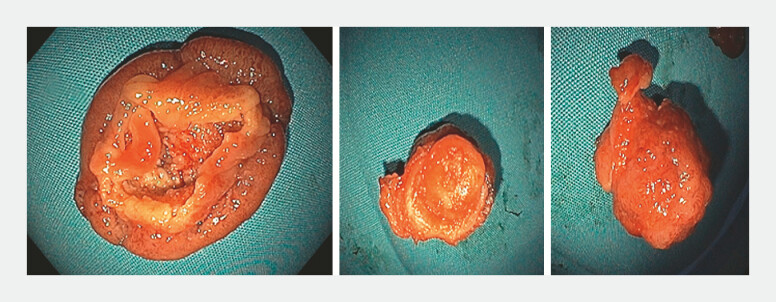
On the left, the cecal lesion successfully resected by uEMR. On the right, the appendiceal stump showing a residual lesion, removed by eFTR. eFTR, endoscopic full-thickness resection; uEMR, underwater endoscopic mucosal resection.

At index colonoscopy, the lesion was classified as type 3a according to Toyonaga’s classification 2 3 showing a pit pattern IIIL–IV according to Kudo classification and a vascular pattern predominantly type 2A and focally 2B according to JNET classification. Appendiceal stump was not identified. In light of the patient’s history of appendectomy and to achieve en bloc resection while minimizing the risk of complications, resection was performed using underwater endoscopic mucosal resection 4 . After resection, residual tissue was identified within the appendiceal stump ( Fig. 1 ). Given the location 5 , endoscopic full-thickness resection was performed to obtain complete excision of the remaining tissue ( Fig. 2 ). The key steps of the procedure are shown in Video 1 .

Underwater EMR of a LST-NG of the cecum. EMR, endoscopic mucosal resection; LST-NG, non-granular laterally spreading tumor.Video 1

**Fig. 3:**
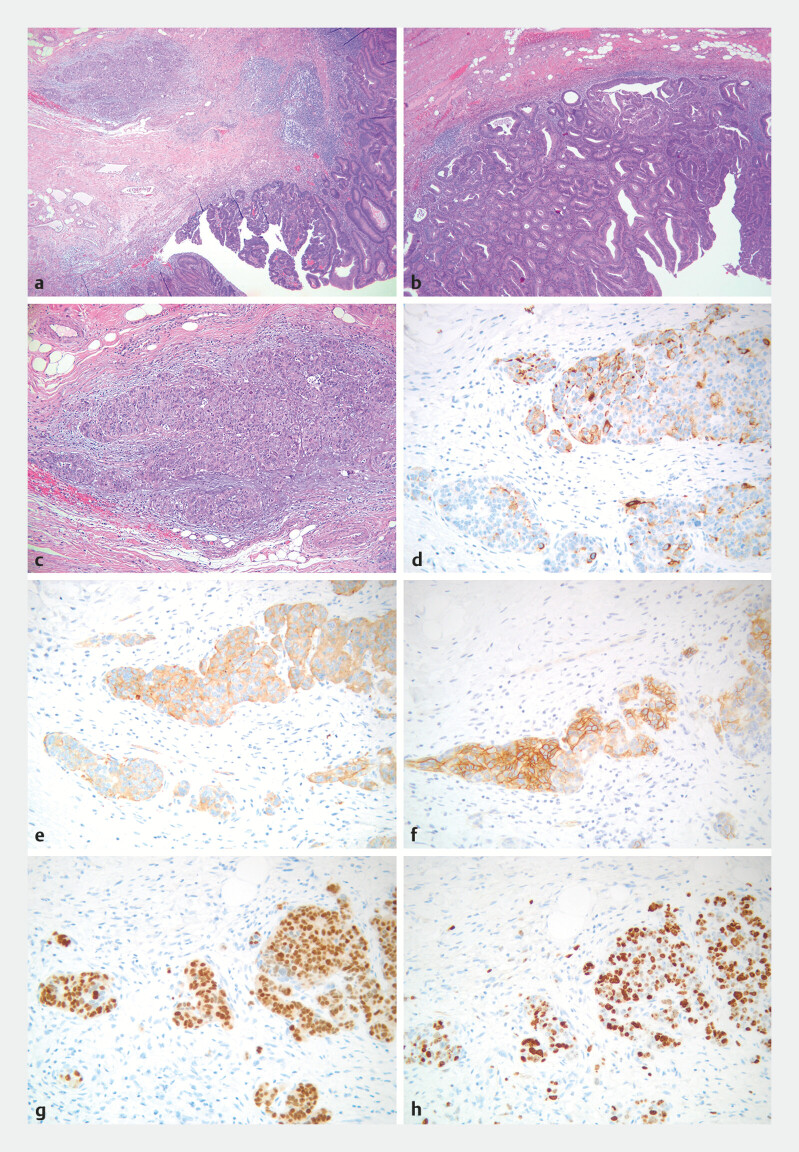
Representative images of the reported tumours. NEN (top left) and the sessile serrated lesion/polyp (right) with a cancerized zone (bottom right) are shown at low magnification ( **a** ). **b** At higher magnification, the sessile serrated lesion/polyp, with its typical dilatation of the deep part of some crypt, and **c** NEN composed of solid nests of epithelioid atypical cells. **d–g** NEN immunoreactivity for chromogranin, synaptophysin, SSTR2A and CDX2, respectively. **h** The high proliferative index of NEN highlighted by Ki67. NEN, neuroendocrine neoplasm.

Histopathological examination revealed distinct entities between the two specimens. The cecal specimen revealed a sessile serrated lesion with focal intramucosal areas of low-grade conventional intestinal-type adenocarcinoma. In contrast, the appendiceal stump specimen demonstrated a high-grade neuroendocrine neoplasm (NEN) with a Ki-67 index exceeding 60%, consistent with large cell neuroendocrine carcinoma. The neoplasm had infiltrated the superficial submucosa (pT1, Fig. 3 ). Both resections achieved negative margins (R0). After multidisciplinary discussion, the patient underwent surgery, which confirmed negative lymph nodes and margins. Postoperative imaging (68Ga-DOTATOC positron emission tomography /computed tomography) showed no residual or metastatic neuroendocrine disease. Due to the high-grade nature of the neuroendocrine neoplasm, adjuvant therapy with somatostatin analogs was initiated, despite radical resection.

This case reports the rare coexistence of distinct histopathological entities and, beyond technical success, demonstrates the importance of advanced endoscopic expertise, allowing complete resection through a tailored and timely switching when required.

Endoscopy_UCTN_Code_CCL_1AD_2AB
